# Computed tomography-guided fine needle aspiration cytology of solitary pulmonary nodules suspected to be bronchogenic carcinoma: Experience of a general hospital

**DOI:** 10.4103/0970-9371.66691

**Published:** 2010-01

**Authors:** Sumana Mukherjee, Gautam Bandyopadhyay, Aparna Bhattacharya, Ritu Ghosh, Gopinath Barui, Rupam Karmakar

**Affiliations:** Pathology, Bankura Sammilani Medical College, P.O. Kenduadihi, Dist. Bankura, West Bengal - 722 102, India; 1 Pathology, R. G. Kar Medical College and Hospital, West Bengal University of Health Sciences, 1, Khudiram Bose Road, Kolkata - 700 004, India; 2 Preventive and Social Medicine, R. G. Kar Medical College and Hospital, West Bengal University of Health Sciences, 1, Khudiram Bose Road, Kolkata - 700 004, India

**Keywords:** Bronchogenic carcinoma, computed tomography, fine needle aspiration cytology

## Abstract

**Background:**

Fine needle aspiration cytology (FNAC) may be diagnostic in candidates with indeterminate solitary pulmonary nodules (SPNs) suspicious of bronchogenic carcinoma.

**Aims:**

The study was performed to evaluate the usefulness of computed tomography (CT)-guided FNAC in our centre.

**Materials and Methods:**

All the cases had a strong clinical suspicion of lung cancer, negative bronchoscopy, negative sputum cytology for malignant cells and acid fast bacilli. A thorough radiological evaluation was made to rule out primary malignancy elsewhere.

**Results:**

A total of 94 patients were studied in one year. May-Grünwald-Giemsa stain was used for the smears. The cytological diagnosis was correlated with clinical-radiological follow-up and biopsy to arrive at a final diagnosis. The procedure had a high sensitivity and specificity. Chi-square test was used to calculate statistical significance. Tumor of more than three centimeter and immediate cytological assessment significantly increased the yield. Review of slides added two cases of malignancy that were missed initially. There were very few complications.

**Conclusions:**

CT-guided FNAC was an accurate and safe procedure for SPNs.

## Introduction

Lung cancer is usually suspected on the basis of an abnormal radiographic imaging study, often in conjunction with symptoms caused by either local or systemic effects of the tumor. The modality selected to diagnose a suspected lung cancer is based on the size and location of the primary tumor in the lung, the presence of potential metastatic spread and the anticipated treatment plan.[1] Computed tomography (CT)-guided fine needle aspiration cytology (FNAC) of suspicious lung masses is a widely accepted and simple diagnostic method of relatively low cost. In patients with lung cancer that is inoperable owing to local factors or the patient’s general condition, FNAC confirms the diagnosis and reveals the tumor type. This is useful in deciding the therapeutic approach in patients in whom results of bronchoscopy and sputum cytological study are not diagnostic. In candidates for surgery with indeterminate solitary pulmonary nodule (SPN) without clear radiologic signs of malignancy or benignity, findings from FNAC may be diagnostic.[2] We performed a study to evaluate the usefulness of CT-guided FNAC in our centre in the diagnosis of lung nodules suspicious of bronchogenic carcinoma as this minimally invasive procedure could be a basis for treatment.

## Materials and Methods

A SPN, suspected to be bronchogenic carcinoma, was defined as a solid, roundish, peripheral (outer half of parenchyma) lung mass in a subject who was more than 20 years of age, with strong clinical suspicion of lung cancer, normal sputum cytology, normal bronchoscopy and sputum for acid fast bacilli negative for three consecutive days. All cases were thoroughly evaluated radiologically to exclude primary malignancy in any site other than lung.

A total of 94 patients met the defined criteria and underwent CT-guided FNAC between December 2006 and December 2007. CT was used before the FNAC to measure the density of the lesion, to accurately localise it and to plan the approach. Patients underwent CT without contrast enhancement in either prone or supine position. Patient positioning was based on the shortest distance from the lesion to the visceral surface, except when there were overlying skeletal structures or large pulmonary vessels to be crossed. Images were obtained through the region of interest by using a section thickness of three mm.

All the patients were aspirated using a 22G disposable spinal needle. The depth from the skin to the lesion periphery was measured by using the CT images and the appropriate length was inserted. After needle insertion, CT was used to confirm the adequacy of needle position. Breath holding was limited to when the needle was being inserted and normal breathing was encouraged at other times. The samples were then obtained by a cytopathologist using aspiration with a 20 ml syringe in half of the cases and 10 ml syringe in the other half. Four to six short jabs were made into the mass, with care taken to biopsy the periphery as well as the centre of the lesion. Immediate cytological assessment was performed by an on-site cytopathologist, which determined the need for a second or a third pass, provided the patient remained asymptomatic and no pneumothorax or hemoptysis occurred. The smears were air dried for May-Grünwald-Giemsa staining and fixed in methanol for hematoxylin and eosin staining.

A repeat slice in the area of interest was taken to rule out pneumothorax. If any amount of pneumothorax developed, the patient was kept under observation for 24 hours and chest radiograph was carried out after 24 hours to rule out any subsequent development of pneumothorax. The final diagnosis of each lesion was determined from examination of surgical specimen using tru-cut needle or by bronchoscope and therapeutic response; similar diagnosis was made elsewhere in the body and from clinical follow-up (minimum period of 15 months). A comparison was made between the cytological diagnosis and the final diagnosis. The results of this procedure (Group A) were compared with a group of 72 patients (Group B) who had undergone the same procedure between 2004 and 2005, but in the absence of immediate cytological assessment by on-site cytopathologist, all other parameters remaining the same. The two groups were compared for the evaluation of the effect of immediate cytological assessment on accuracy. The material obtained was fixed on slides using a spray preparation with polyethylene glycol base. Immediate cytological examination was performed for adequacy of material after staining the slides with methylene blue using a light microscope.

The study group was also evaluated for other factors affecting accuracy, like tumor size, suction used, review of slides and development of complications. The statistical significance was calculated by using the chi-square test. *P*-value <0.05 was considered to indicate statistical significance.

## Results

**Figure 1:**
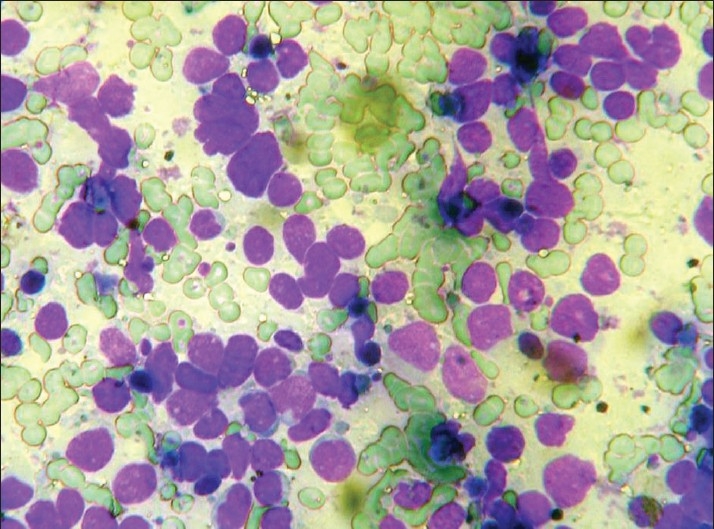
Small cell carcinoma of lung: CT-guided FNAC smears (MGG, ×400)

**Figure 2:**
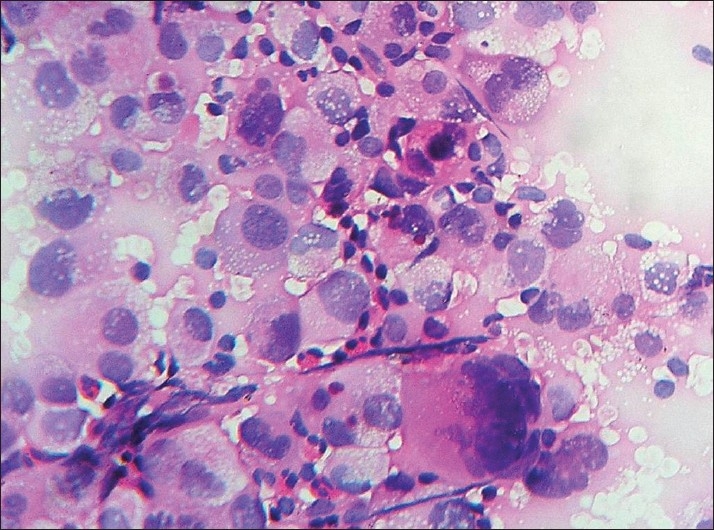
Poorly differentiated carcinoma of lung: CT-guided FNAC smears (H and E, ×400)

Of the 94 cases in this study, 90 cases were proved to be malignant at final diagnosis. Among the malignant cases, most patients (76%) were in the age group of 40–70 years, and most were males (85%). There was no false positives for malignancy. Only two cases that were malignant were missed at cytology. Hence, there was 97.7% sensitivity and 100% specificity for CT-guided FNAC as a diagnostic procedure [Table 1]. Histological typing was available in 88 cases and was compared with the cytological subtype. Overall accuracy for cytological subtyping [Figures 1 and 2] was 95% [Table 2]. Chi-square test was used to calculate the effect of various factors on the accuracy of the results obtained [Table 3]. Lesions more than or equal to three cm in size gave significantly higher yield than lesions less than three cm in size. Immediate cytological assessment significantly increased the number of cases with adequate material. “Adequate sample” was one that permitted a specific diagnosis. If a specific diagnosis was not possible, a high cellularity was considered adequate. Of the 72 patients in Group B, 11 cases were inadequate and could not be included in the calculation, 48 cases were true positive, seven were true negative and six cases were false negative. Variation in the suction used had no significant effect on the yield. Blind review of all slides by another observer revealed two additional cases of malignancy. These cases were diagnosed initially as negative and were reclassified at review as unsatisfactory. Repeat FNAC in these cases proved them to be positive for malignancy.

Only four cases developed complications. Three were cases of minor pneumothorax and there was one case with hemoptysis.

**Table 1:** Comparison of statistical analysis in the two groups

Statistical parameter	Group A (%)	Group B (%)
Sensitivity	97.7	88.8
Specificity	100	100
+ve predictive value	100	100
-ve predictive value	66.67	53.8

**Table 2:** Accuracy of cytological subtyping

Subtype	Cytological diagnosis	Histological diagnosis
Non-small cell carcinoma lung		
Squamous cell carcinoma	7	8
Adenocarcinoma	40	40
Small cell carcinoma lung	25	27
Poorly differentiated carcinoma lung	7	8
Benign lesions	4	4
Lymphoma	1	1

**Table 3:** Effect of various factors on diagnostic accuracy

	No. of cases	Positive yield/cases with adequate material	*P* value
Size of lesion			1.6298E-08 1.6298E-08 (extremely significant)
< 3 cm	39	35	
> 3 cm	55	53	
Presence of on-site cytopathologist			2.04503E-36 (extremely significant)
Study group A	94	94	
Study group B	72	61	

## Discussion

In our study, there was good correlation between the original cytological diagnosis by CT-guided FNAC and the final clinical–pathological diagnosis. Only two cases were false negative for malignancy and there were no false positives. The high sensitivity and specificity of the study agreed with that of many workers in this field.[3–11] The huge success of CT-guided FNAC in this study can be attributed to the experience of the physicians involved, the high index of suspicion in all the cases selected and all were easily accessible by CT-guided FNAC. Thus, our pre-test probability was higher for a diagnosis of malignancy.

Non-small cell carcinomas predominated, similar to the experience of other workers.[8,12] The overall accuracy of diagnosing small cell versus non-small cell lung cancer at cytology in this study was 96%. The high accuracy of distinction between small cell and non-small cell lung cancer in our study matched with other studies.[1,13–15] Cytological diagnosis of non-small cell lung cancer [Figure 2] is more reliable (misclassification in 2% of the cases) than cytological diagnosis of small cell lung cancer [Figure 1] (misclassification in 7% of the cases).

Lesions more than or equal to three cm in size gave significantly higher yield than lesions less than three cm in size. Other workers have also observed that larger lesions increased the diagnostic accuracy. Studies have described the accuracy in diagnosis of lesions more than 1.5 cm in diameter, more than two cm in diameterand more than three cm in diameter.[7,16–20] Most of our cases presented late, with relatively larger lesions.

Immediate assessment of the specimen by on-site cytopathologist with further passes made when necessary has been shown by previous workers to improve the adequacy rates of the technique.[9,2,21] When we introduced immediate cytological assessment in our study, our adequacy rates rose significantly.

The importance of review of the slides has been cited by Tan *et al*.[22] We found that the review was especially important in the cases diagnosed as negative for malignancy. We also found that the review of slides by a second expert followed by a consensus diagnosis by both increased the accuracy.

The rate of development of pneumothorax reported in the literature is variable.[6,7,11,17,23,24] Our strikingly low rate of complication can be explained by immediate cytological assessment requiring minimum number of passes to be made and peripheral lesions relatively large in size.

The study concludes that CT-guided FNAC is an accurate and safe method with high sensitivity and specificity for SPNs. It can subclassify the type of bronchogenic carcinoma and the vast majority of lung malignancies can be confidently diagnosed with cytomorphological characterization in the right clinical context. Unsatisfactory and other non-malignant smears should be reviewed and reaspirations may be necessary.
